# The Insanity of Over-Exertion of the Brain

**Published:** 1895-06

**Authors:** 


*The Insanity of Over-exertion of the Brain.* By J. BATTY TUKE, M.D. Pp. viii., 66. Edinburgh: OLIVER AND BOYD. [1894.]

This monograph consists of the five Morison Lectures delivered before the Royal College of Physicians of Edinburgh last year. Dr. Batty Tuke has long been known as a scientific physician, and it may be stated at once that these lectures will enhance his already high reputation.

At the outset he suggests that insanity has been too long regarded from the purely psychological, to the neglect of the physiological, point of view. This he justly condemns as an antiquated and unscientific basis, leading to faulty views and nomenclature. Nowadays this last should be etiological rather than symptomatic. Insanity is merely a symptom, not an entity in itself.

In Lecture I. the anatomy of a cerebral convolution according to the most recent observers is given. This is illustrated and greatly simplified by an admirable diagram founded on the description of Ramón y Cajal, which further assists us to "visualise" the morbid changes Dr. Tuke traces in the condition forming the subject of his lectures. Lecture II. describes the results of over-exertion and hyperæmia of the cortex. This is illustrated by plates showing the effects of electrical stimulation on nerve-cells after Dr. C. F. Hodge. In Lecture III., in which further morbid changes are sketched, Dr. Tuke modifies his former views on "miliary sclerosis," and admits that this change arises from aggregations of the myelin around nerve-fibre. Lecture IV. is concerned with the somatic symptoms of over-exertion. In passing rapidly over what will repay careful perusal, we notice the following statements:—Loss of tone of the digestive apparatus is an almost invariable prodromal symptom. For all practical purposes, peripheral irritation may be dismissed from the list of producers of insanity, otherwise many cases of insanity would be found in surgical and general hospitals. Insanity is not directly occasioned by amenorrhœa or any other uterine or ovarian disease, and therefore operation on these organs with the idea of relieving mental symptoms is not legitimate. Insanity is never the consequence of disease of individual organs, but rather of diathesis or cachexia, *e.g.* syphilis, tuberculosis, &c. From this last proposition we must dissent. In Lecture V. the rules of treatment for this class of cases are laid down.

This book will make its mark in the literature of the Nervous System of the present day. It demands and well deserves thoughtful study. If some of its statements are not yet proved to demonstration, and may require future modification, it is conceived in a thoroughly scientific spirit, and too much is not attempted. "The mechanics of psychical processes are matters of surmise and theory: all we can say is, that mental action is a function of connections. Interrupt these, modification of function must ensue."

